# Norms for Automatic Estimation of Hippocampal Atrophy and a Step Forward for Applicability to the Italian Population

**DOI:** 10.3389/fnins.2021.656808

**Published:** 2021-06-28

**Authors:** Silvia De Francesco, Samantha Galluzzi, Nicola Vanacore, Cristina Festari, Paolo Maria Rossini, Stefano F. Cappa, Giovanni B. Frisoni, Alberto Redolfi

**Affiliations:** ^1^Laboratory of Neuroinformatics, IRCCS Istituto Centro San Giovanni di Dio Fatebenefratelli, Brescia, Italy; ^2^Laboratory of Alzheimer’s Neuroimaging and Epidemiology - LANE, IRCCS Istituto Centro San Giovanni di Dio Fatebenefratelli, Brescia, Italy; ^3^National Center for Disease Prevention and Health Promotion, National Institute of Health, Rome, Italy; ^4^Department of Neuroscience and Neurorehabilitation, IRCCS San Raffaele Pisana, Rome, Italy; ^5^IRCCS Mondino Foundation, Pavia, Italy; ^6^IUSS Cognitive Neuroscience (ICoN) Center, University School for Advanced Studies, Pavia, Italy; ^7^Memory Clinic and LANVIE - Laboratory of Neuroimaging of Aging, University Hospitals and University of Geneva, Geneva, Switzerland

**Keywords:** magnetic resonance imaging, automatic segmentation tools, normative distribution, hippocampal volume, aging

## Abstract

**Introduction:**

Hippocampal volume is one of the main biomarkers of Alzheimer’s Dementia (AD). Over the years, advanced tools that performed automatic segmentation of Magnetic Resonance Imaging (MRI) T13D scans have been developed, such as FreeSurfer (FS) and ACM-Adaboost (AA). Hippocampal volume is considered abnormal when it is below the 5th percentile of the normative population. The aim of this study was to set norms, established from the Alzheimer’s Disease Neuroimaging Initiative (ADNI) population, for hippocampal volume measured with FS v.6.0 and AA tools in the neuGRID platform (www.neugrid2.eu) and demonstrate their applicability for the Italian population.

**Methods:**

Norms were set from a large group of 545 healthy controls belonging to ADNI. For each pipeline, subjects with segmentation errors were discarded, resulting in 532 valid segmentations for FS and 421 for AA (age range 56–90 years). The comparability of ADNI and the Italian Brain Normative Archive (IBNA), representative of the Italian general population, was assessed testing clinical variables, neuropsychological scores and normalized hippocampal volumes. Finally, percentiles were validated using the Italian Alzheimer’s disease Repository Without Borders (ARWiBo) as external independent data set to evaluate FS and AA generalizability.

**Results:**

Hippocampal percentiles were checked with the chi-square goodness of fit test. *P*-values were not significant, showing that FS and AA algorithm distributions fitted the data well. Clinical, neuropsychological and volumetric features were similar in ADNI and IBNA (*p* > 0.01). Hippocampal volumes measured with both FS and AA were associated with age (*p* < 0.001). The 5th percentile thresholds, indicating left/right hippocampal atrophy were respectively: (i) below 3,223/3,456 mm^3^ at 56 years and 2,506/2,415 mm^3^ at 90 years for FS; (ii) below 4,583/4,873 mm^3^ at 56 years and 3,831/3,870 mm^3^ at 90 years for AA. The average volumes computed on 100 cognitively intact healthy controls (CN) selected from ARWiBo were close to the 50th percentiles, while those for 100 AD patients were close to the abnormal percentiles.

**Discussion:**

Norms generated from ADNI through the automatic FS and AA segmentation tools may be used as normative references for Italian patients with suspected AD.

## Introduction

Normal brain aging can be defined as a typical biological process of the elderly population, characterized by reduction of cerebral volume without severe affection of cognitive functions (Fjell et al., 2014). However, a universally accepted pathologic cut-off between physiological and abnormal aging of the brain does not exist.

Many neurodegenerative diseases are characterized by specific structural changes visible using anatomical magnetic resonance imaging (MRI). The main issue is that the brain MRI scans are usually rated by neuroradiologists with subjective qualitative visual evaluations based on their own experience and expertise about how the normal brain should appear (Vernooij et al., 2019). The community of expert neuroradiologists (who read at least more than 500 brain scans per year) believes that accuracy and reproducibility drop dramatically in young or non-expert neuroradiologists (McCarron et al., 2006), resulting in waste of resources and inappropriate diagnosis.

Among brain structures, hippocampal volume (HV) is one of the key biomarkers in the diagnostic assessment of Alzheimer’s Dementia (AD) (Frisoni et al., 2010). Atrophic changes start in the early stages of the development of AD, some years before the symptoms begin to manifest (Apostolova et al., 2010). The need for a definition of what a “normal” hippocampal structure should be, has been further enhanced by the inclusion of the hippocampal volume as marker of neurodegeneration in the National Institute on Aging and Alzheimer’s Association (NIA-AA) criteria for the diagnosis of AD (Albert et al., 2011; McKhann et al., 2011).

The accurate and reproducible segmentation of the hippocampal borders via a precise volumetric quantification represents a significant advancement in comparison to subjective assessment. Manual segmentation is considered the gold standard and, more recently, automatic segmentation methods were used to get as close as possible to results gathered via manual delineation (Dill et al., 2015). Over the last 5 years, in many research centers the labor-intensive hand-tracing segmentation of the hippocampal region, requiring a large amount of time and trained experts to be completed, has been replaced by advanced tools that perform an automatic segmentation of T1-weighted 3D (T13D) MRI. These tools can compute the hippocampal volume in a reduced period of time and with minimal inter-operator differences (Inglese et al., 2015; Maglietta et al., 2016; Bosco et al., 2017). They save time and money by approximating the atrophy measures obtained with manual tracing (Cover et al., 2018; Schmidt et al., 2018). Moreover, automatic tools allow definition of normative data and relative cut-off, comparative analysis during follow-up, reduce the variability and allow the parallel processing of multiple images. These advances facilitate the usage of the hippocampal biomarker in national as well as in international large-scale clinical and observational studies.

Many automatic segmentation tools have been proposed so far. They use different anatomic libraries, pipelines, segmentation protocols and differ in the computational time. Two popular automatic tools giving a reliable quantification of the hippocampal volume are: FreeSurfer (FS) (Morey et al., 2009; Fischl, 2012), based on probabilistic atlas and voxel labeling via spatial localization priors and intensity features; and the Auto Context Model—Adaboost (AA), based on a weak-learner algorithm exploiting the extraction of thousands of features in a hippocampal bounding-box, such as: image intensity, tissue classification maps, gradient filters, curvatures, Haar filters of different sizes, neighborhood features (Morra et al., 2008).

The shrinkage process of hippocampal volume is progressing with age both in cognitively intact persons and in AD patients, but at different rates (Barnes et al., 2009). Also gender and head size may influence the hippocampal volume. The influence of the latter two factors can be reduced if the hippocampal values are normalized for intracranial volume (TIV) (Scahill et al., 2003). In this way, the hippocampus of a subject can be compared with that of a reference population of persons with normal cognition, and the volumetric information can be translated into an age-specific percentile. When the volumetric value of the hippocampus is below the 5th percentile, it can be considered as abnormal and may be related to the presence of cognitive impairment.

The definition of normality is of course a complex issue and, obviously, hundreds of normal subjects must be used for the definition of norms. The biggest problem is that large numbers of MRI of cognitively intact persons, representative of the general population, and carried out with nearly identical technological parameters, are very difficult to collect. Historically, only one multicentric initiative in Italy, called Italian Longitudinal Study on Aging (ILSA), collected comprehensive data from a population-based cohort (Maggi et al., 1994) but it lacked brain scans. To the best of our knowledge, no single center in Italy has sufficiently large population-based data ready to be exploited using scans easily exportable from Picture Archiving and Communication Systems (PACS). A convenient alternative is to use scans from people who underwent MRI in large public observational studies, such as Alzheimer’s Disease Neuroimaging Initiative (ADNI), and who were labeled as healthy normal controls. If this group shows clinical and neuropsychological characteristics similar to those of the Italian general population, this could imply that their structural brain features can be regarded as representative of the general population.

In this study we aimed: (i) to set norms for both FS and AA hippocampal volumetry using data from ADNI database (Petersen et al., 2010); (ii) to assess the comparability between the US population from ADNI and the Italian population represented by the Italian Brain Normative Archive (IBNA); (iii) to report any differences between the two automatic tools using an independent large dataset of Italian patients, the Italian Alzheimer’s Repository Without Border (ARWiBo), representative of the entire AD spectrum.

## Materials and Methods

### Study Design

The normative percentiles for each algorithm were calculated from the ADNI normative population. Then, the features of the ADNI normative population were compared to IBNA data set (Riello et al., 2005; Galluzzi et al., 2009), including clinical, neuropsychological and volumetric variables. Norms were further assessed with the independent ARWiBo data set (Neu et al., 2017). The percentiles created are made available in neuGRID^1^, an on-line e-infrastructure providing tools for automatic quantification of hippocampal volume (Redolfi et al., 2013).

### Data

The group used to generate the percentiles included 545 cognitively intact healthy controls (CN) selected among those enrolled in ADNI studies who had at least a volumetric scan at baseline. T13D MRI sequences with artifacts precluding hippocampal measurements were discarded. Hippocampal segmentations quality control was conducted by experienced neuroscientists (SD, AR) who inspected slice by slice the hippocampal masks derived with FS and AA. Subjects showing over or under-segmentation errors were discarded. This resulted in two numerically different populations, i.e., 532 subjects for FS and 421 subjects for AA (see Supplementary Table 1 for the complete subjects lists).

ADNI normative data set was collected from the Imaging Data Archive (IDA) web-portal of the Laboratory of NeuroImaging (LONI)^2^.

The Italian general population data set used to test the transferability of ADNI percentiles to the Italian population, focusing on clinical, neuropsychological and volumetric variables, was IBNA. IBNA is composed by 483 CN subjects who underwent brain scan at the Neuroradiology Unit of the “Città di Brescia” Hospital, Brescia, from March 2001 to May 2006. Reasons to perform MRI were other than cognitive impairment or other suspected organic brain disease. Subjects were enrolled if brain scan was judged as normal by the neuro-radiologist based on visual assessment and were excluded if they showed neurological deficits. Local ethics committee approved the study.

Then, to further validate the percentile curves of FS and AA with an independent Italian data set we selected a substantial group of 100 CN, 100 mild cognitive impairments (MCI) and 100 AD subjects (ranging from 56 to 90 years and with an isotropic T13D MRI) from the independent ARWiBo data set. ARWiBo is a population based cross-sectional data set including more than 2,500 patients from 20 to 92 years old, enrolled in Brescia (Italy) and nearby areas (Archetti et al., 2019). The data set contains socio-demographic, clinical, genetic, biological information and T13D images (Frisoni et al., 2009).

### Clinical, Neuropsychological, and Socio-Demographic Assessments

Clinical and neuropsychological assessment tests administered in ADNI, IBNA, and ARWIBO are reported in Supplementary Table 2.

Clinical variables of ADNI were compared to 96 IBNA subjects whose characteristics were reported to be similar to ILSA and, consequently, representative of the general Italian population (Galluzzi et al., 2009). The variables examined were the ones that most commonly affect the physical health and the cognitive status of elderly people. Therefore hypertension, diabetes, heart diseases, severe obesity [calculated as Body Mass Index (BMI) > 40], CDR and depression scales were compared between ADNI and IBNA. Among the neuropsychological tests, Mini Mental State Examination (MMSE), Trail Making Test A (TMT-A), Trail Making Test B (TMT-B), verbal fluency, logical memory, clock drawing, digit span, and Rey auditory verbal learning were compared between ADNI and IBNA. To ensure comparability among the data sets and to overcome the protocol difference in the administration of neuropsychological tests, the comparison was performed by computing and comparing the z-scores or t-scores based on the group of CN of each data set. Because of the large discrepancy in age and education between ADNI and IBNA data sets, and considering the influence of these variables on the final test scores, the neuropsychological comparison was conducted on a subpopulation selected considering: the presence of T13D MRI, the intersection between the cohorts in the age range between 55 and 80 years, education between 5 and 19 years, a random reduction of ADNI cases to limit its oversampling, a comparable proportion of Apolipoprotein E ε4 (ApoE4) carriers (see Table 1). Furthermore, in all the comparison performed, subjects with missing values in the studied variables were excluded resulting in different sample sizes.

**TABLE 1:** Clinical, neuropsychological, morphological features comparisons between IBNA and ADNI data sets.

	IBNA	ADNI FS	*P*-value	ADNI AA	*P*-value
**Clinical (full samples)**					
Age	72 ± 5 (n = 96)	74 ± 6 (*n* = 532)	0.009	73 ± 6 (*n* = 421)	0.047
Education	8 ± 4 (*n* = 88)	16 ± 3 (*n* = 516)	<0.001	16 ± 3 (*n* = 409)	<0.001
ApoE4 carriers	4 (8%) (*n* = 48)	100 (27%) (*n* = 365)	0.004	70 (26%) (*n* = 265)	0.007
Gender, female	64 (67%) (*n* = 96)	290 (55%) (*n* = 532)	0.027	229 (54%) (*n* = 421)	0.029
Ethnicity	100% White	White 88% African 6% Asian 2% Other 4%	–	White 89% African 5% Asian 2% Other 4%	–
Hypertension	50 (52%) (*n* = 96)	229 (43%) (*n* = 532)	0.101	174 (41%) (*n* = 421)	0.055
Diabetes	7 (7%) (*n* = 96)	31 (8%) (*n* = 375)	0.754	23 (8%) (*n* = 272)	0.720
Heart disease	29 (30%) (*n* = 96)	97 (26%) (*n* = 375)	0.391	76 (28%) (*n* = 272)	0.672
Severe obesity	1 (1%) (*n* = 94)	8 (1%) (*n* = 531)	1	8 (2%) (*n* = 420)	0.899
CDR	0 (100%) 0.5 (0%) 1 (0%) 2 (0%) 3(0%) (*n* = 49)	0 (100%) 0.5 (0%) 1 (0%) 2 (0%) 3(0%) (*n* = 531)	1	0 (100%) 0.5 (0%) 1 (0%) 2 (0%) 3(0%) (*n* = 420)	1
Depression*	0 [−0.20; +0.20] (*n* = 96)	0 [−0.08; +0.08] (*n* = 532)	0.587	0 [−0.10; +0.10] (*n* = 421)	0.771
**Neuropsychological (matched samples)**					
Age	66 ± 7 (*n* = 64)	70 ± 3 (*n* = 68)	<0.001	70 ± 3 (*n* = 55)	<0.001
Education	11 ± 3 (*n* = 64)	13 ± 1 (*n* = 68)	<0.001	13 ± 1 (*n* = 55)	<0.001
ApoE4 carriers	6 (13%) (*n* = 46)	13 (26%) (*n* = 49)	0.166	10 (28%) (*n* = 36)	0.165
MMSE	0 [−0.24; +0.24] (*n* = 64)	0 [−0.24; +0.24] (*n* = 68)	0.119	0 [−0.26; +0.26] (*n* = 55)	0.142
TMT-A	0 [−0.24; +0.24] (*n* = 64)	0 [−0.24; +0.24] (*n* = 68)	0.951	0 [−0.26; +0.26] (*n* = 55)	0.972
TMT-B	0 [−0.24; +0.24] (*n* = 64)	0 [−0.24; +0.24] (*n* = 68)	0.940	0 [−0.26; +0.26] (*n* = 55)	0.960
TMT B-A	0 [−0.24; +0.24] (*n* = 64)	0 [−0.24; +0.24] (*n* = 68)	0.752	0 [−0.26; +0.26] (*n* = 55)	0.817
Verbal fluency (phonemic)	0 [−0.24; +0.24] (*n* = 64)	0 [−0.32; +0.32] (*n* = 38)	0.887	0 [−0.33; +0.33] (*n* = 36)	0.771
Verbal fluency (semantic)	0 [−0.24; +0.24] (*n* = 64)	0 [−0.24; +0.24] (*n* = 68)	0.687	0 [−0.26; +0.26] (*n* = 55)	0.626
Logical memory	0 [−0.28; +0.28] (*n* = 48)	0 [−0.24; +0.24] (*n* = 68)	0.648	0 [−0.26; +0.26] (*n* = 55)	0.675
Clock drawing test	0 [−0.29; +0.29] (*n* = 47)	0 [−0.24; +0.24] (*n* = 68)	0.936	0 [−0.26; +0.26] (*n* = 55)	0.972
Digit span forward	0 [−2.31; +2.31] (*n* = 9)	0 [−2.05; +2.05] (*n* = 30)	0.960	0 [−2.10; +2.10] (*n* = 19)	0.655
Rey Auditory Verbal Learning Test—Immediate	0 [−0.26; +0.26] (*n* = 55)	0 [−0.24; +0.24] (*n* = 68)	0.990	0 [−0.26; +0.26] (*n* = 55)	0.948
Rey Auditory Verbal Learning Test—Delayed	0 [−0.26; +0.26] (*n* = 55)	0 [−0.24; +0.24] (*n* = 68)	0.663	0 [−0.26; +0.26] (*n* = 55)	0.825
Rey Auditory Verbal Learning Test—Recognition	0 [−2.05; +2.05] (*n* = 28)	0 [−1.99; +1.99] (*n* = 67)	0.931	0 [−2.01; +2.01] (*n* = 55)	0.973
**Morphological (matched samples)**					
Left HV FS	3,638 ± 328 (*n* = 64)	3,611 ± 401 (*n* = 68)	0.077	–	–
Right HV FS	3,739 ± 341 (*n* = 64)	3,729 ± 406 (*n* = 68)	0.884	–	–
Left HV AA	5,314 ± 460 (*n* = 64)	–	–	5,142 ± 463 (*n* = 55)	0.257
Right HV AA	5,376 ± 454 (*n* = 64)	–	–	5,306 ± 492 (*n* = 55)	0.533

Values denote means ± standard deviation or number (%). The 95% Confidence Intervals (CI) of z-scores or t-scores (when at least one group size was *n* < 30) are reported in square brackets. Clinical *p*-values denote the significance on Chi-square test for the dichotomous variables and Mann-Whitney test for continuous ones. *P*-values of the neuropsychological and volumetric comparison are obtained with Mann-Whitney test or ANOVA considering the data distribution. n, sample size; IBNA, Italian Brain Normative Archive; ADNI, Alzheimer’s Disease Neuroimaging Initiative; FS, FreeSurfer; AA, ACM-Adaboost; CDR, Clinical Dementia Rating scale. MMSE, Mini-Mental State Examination; TMT-A, Trail Making Test (part A); TMT-B, Trail Making Test (part B); TMT B-A, Trail Making Test (part B minus part A); HV, Hippocampal Volume expressed in mm^3^.

*Geriatric Depression Scale (GDS) or Brief Symptom Inventory (BSI) scale.

### MR Imaging

ADNI brain MR images selected were T13D magnetization-prepared rapid acquisition with gradient echo (MPRAGE) sequences acquired with a field strength of 1.5 (*FS* = 222; *AA* = 131) or 3 Tesla (*FS* = 310; *AA* = 290). MPRAGE scans^3^ were acquired in the sagittal plane with isotropic 1 mm voxel size and with a gradient echo 3D technique optimized and harmonized for the three main scanner manufacturers (i.e., PHILIPS, GE, SIEMENS).

IBNA MRIs were acquired exclusively with a PHILIPS Gyroscan scanner at 1.0 Tesla. The T1-weighted scan was acquired in the sagittal plane with a gradient echo 3D technique as follows: *TR* = 20 ms, *TE* = 5 ms, flip angle = 30°, acquisition matrix 256 × 256, slice thickness = 1.3 mm.

ARWiBo scans were acquired with a PHILIPS Gyroscan scanner at 1.0 Tesla or with a GE Signa HDx at 1.5 Tesla and an Inversion Recovery Spoiled Gradient Echo as follows: *TR* = 12 ms, *TE* = 5 ms, TI = 600 ms; flip angle = 8°, acquisition matrix 256 × 256, slice thickness = 1 mm.

### Hippocampal Volume

Right and left hippocampal volumes for the subpopulations selected were obtained with FS v.6.0 and AA. FS is a pipeline for the segmentation of brain’s cortical and subcortical structures where each voxel is labeled using a probabilistic atlas (Fischl et al., 2002). The probabilistic atlas was constructed based on a training set of hundreds of manually segmented images by experts of the Massachusetts General Hospital (MGH), Boston, United States. The T13D MR images we analyzed were pre-processed via cross-sectional stream through recon-all script. The volume-based stream is fully described in Fischl et al. (2002, 2004). Finally, hippocampal volumes in native space were normalized in neuGRID to the FS estimated TIV (eTIV) dividing the HV by the subject’s intracranial volume and multiplying the ratio by a reference value of 1,409 ml (Reite et al., 2010) to remove the effect of the head size.

AA is a machine learning tool originally developed at laboratory of Neuro Imaging—University of California Los Angeles (UCLA) to segment the brain hippocampi. It uses a training set of data to develop rules for classifying unseen data. This set consists of 100 T13D MPRAGE MRI and manual tracings (Boccardi et al., 2015) derived by two hippocampus experts from the “harmonized protocol for hippocampal volumetry” project (EADC-ADNI HarP)^4^. We adopted the same leave one out validation strategy reported in Morra et al. (2008) to fine tune the algorithm hyperparameters. AA back-transformed the brain and the hippocampus segmented regions from stereotactic to native space using the FSL convert-xml script. The TIV measurement in the AA pipeline was obtained via the Statistical Parametric Mapping Tool (SPM12)^5^ and, as previously performed in FS, the normalization was accomplished considering the reference intracranial volume of 1,409 ml (Reite et al., 2010).

Supplementary Figure 1 shows a comparison of the hippocampal masks segmented by the two pipelines (FS and AA) on the same MRI.

Test-retest reliability of both tools has been tested on 100 ADNI subjects computing reproducibility errors and Pearson’s correlation (see Supplementary Table 3).

FS and AA volumetric reports generated via neuGRID are available as Supplementary Figures 2, 3.

### Statistical Analysis

Differences in sociodemographic, clinical, neuropsychological and morphological features between data sets (IBNA vs. ADNI) and among subgroups (CN vs. MCI vs. AD) were assessed by analysis of variance (ANOVA), Mann-Whitney test or Kruskal-Wallis test, considering the data distribution and the number of groups, for continuous variables and Chi-squared for dichotomous variables. *Post-hoc* analysis was carried out to test continuous and binary markers differences between the three diagnostic groups of ARWiBo. Tests were two-tailed and the threshold for significance was set at *p* = 0.01.

Multivariate independent component analyses to assess the overall comparability of the subgroups of each data set were computed using MANOVA statistical method along two principal dimensions.

The quantitative effect of education, gender, ApoE4, field strength and vendor has been computed with a Generalized Linear Model (GLM).

As far as the percentile curves are concerned, we tested the distributions that best fitted the hippocampal volumetric data with the “allfitdist” Matlab function (Sheppard, 2012). We assumed a decreasing monotonous trend for both hemispheres and tools. The fit quality was assessed by the chi-square goodness of fit test (“chi2gof” function). Percentile reference curves were created using the Generalized Additive Models for Location, Scale and Shape (GAMLSS). For each age range, specific cut-off values of FS and AA were computed accordingly to the following percentiles: 95th, 90th, 75th, 50th, 25th, 10^*th*^, and 5th for the normalized hippocampal values. The abnormality is represented only when the volume is atrophic, therefore being a one-tailed test, the discrimination threshold considered was equal to the 5th percentile.

Further, the hippocampal volumes of the IBNA population processed with FS and AA (“real volumes”) were compared with those derived from ADNI population norms (“computed volumes”). The individual age from the IBNA subjects was entered in the GAMLSS fitted models of FS and AA and the expected volumes were derived. The difference between real and computed volumes as well as the 95% CI of the difference were estimated. A small difference was taken to denote that the IBNA and ADNI normative populations were similar.

In the ARWiBo cohort, the Cohen’s kappa (κ) coefficient of hippocampal volumes to be under the 5th percentile was investigated in both algorithms.

Finally, linear regression analysis was conducted to assess the relationship between age and hippocampal volumes. FS and AA showed skewed hippocampal distributions therefore they were log-transformed to improve normality prior to analysis.

Chi-squared, Kruskal-Wallis, MANOVA, GLM, GAMLSS, and linear regression tests were executed in R v.3.5.1.

ANOVA, Mann-Whitney and chi-square goodness of fit tests run in Matlab R2016b.

## Results

### Comparison Between Italian and American Data Sets

The ADNI and IBNA characteristics are reported in Table 1.

The IBNA group was younger than the ADNI groups, less educated, with lower prevalence of ApoE4 carriers and with a higher female prevalence. As far as the clinical features are concerned, the IBNA subgroup had a similar prevalence of diabetes, hypertension, heart disease and severe obesity to ADNI subjects. CDR scores in IBNA and ADNI were equal to 0. Finally, the depression scale scores were comparable.

We did not find significant differences in the z-score or t-score for any neuropsychological test between the IBNA subgroup of 64 individuals and FS or AA ADNI subgroups (respectively, 68 and 55 subjects).

Descriptive statistics for morphological measurements are given in Table 1. Both FS and AA showed lower volume in the left hippocampus vs. the right side although not significantly. No significant differences were registered in the volumes between the IBNA and ADNI groups with both tools. The distribution of IBNA volumes can be found in Supplementary Figure 4.

Comparability assessment of the subsamples of each data set considered in Table 1 tested with multivariate statistics (*p* > 0.01) can be found as Supplementary Figure 5.

### Percentile Creation

**FIGURE 1:**
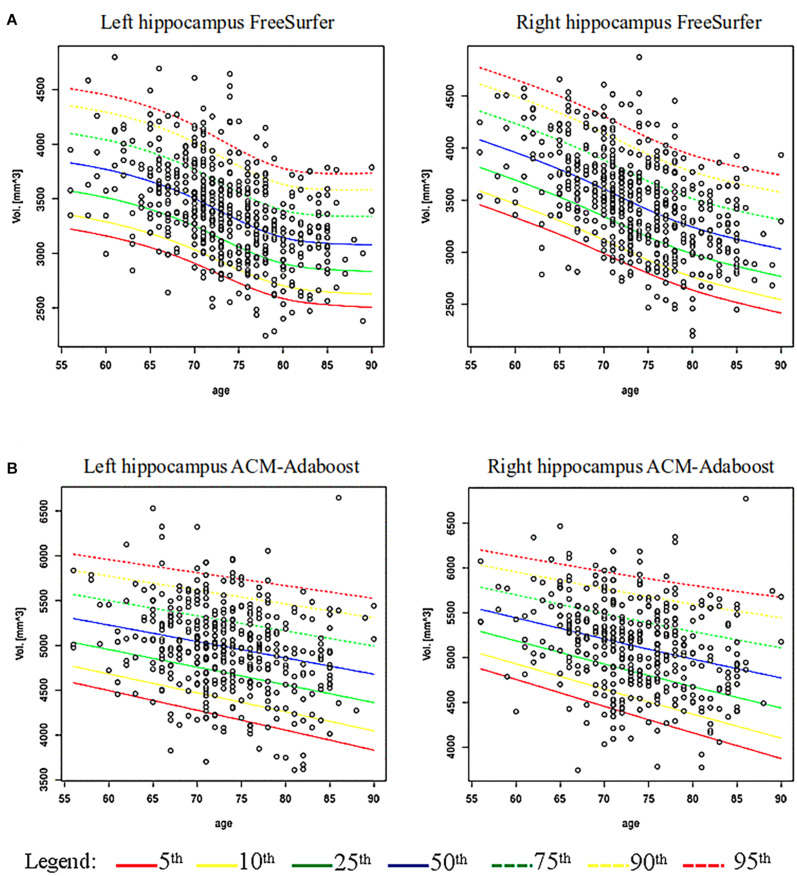
Age-specific percentile distribution of hippocampal volume normalized for total intracranial volume. **(A)** Shows age-specific distribution of hippocampal volumes computed via FreeSurfer (FS) (532 subjects) and its percentiles fitting a Gamma distribution on ADNI data. **(B)** Shows the hippocampal volumes computed via ACM-Adaboost (AA) (421 subjects) fitting a Logistic distribution on ADNI data.

Figure 1 shows age specific percentile distributions for FS and AA based on ADNI datasets. The Gamma distribution best fitted the trend of hippocampal volumes population for FS in relation to age. The fit quality was good, with *p*-value equal to 0.57 for the left hippocampus and 0.21 for the right hippocampus. The logistic distribution best fitted the hippocampal volumes for AA, whose *p*-values were 0.29 and 0.11 for left and right hippocampus, respectively. The number of subjects below each percentile curve were close to the expected value (FS maximum discrepancy: 0.94% for left and 1.13% for right hippocampus; AA maximum discrepancy: 1.65% for left and 2.32% for right hippocampus) and the Chi-square test applied to these percentages showed *p*-values equal to: 0.62, 0.55, 0.12, 0.27. Characteristics of the ADNI subjects with hippocampal volumes under the 5th percentile are reported in Table 2.

**TABLE 2:** Characteristics of ADNI subjects below 5th percentile.

	Left HV < 5th		Right HV < 5th		Left HV and Right HV < 5th	Age	MMSE	ApoE carriers	Family history of dementia
FS	27		23		14	>60	>26	7	20
AA	28		21		10	>60	>28	5	23
		9		5					

*Values reported the number of subjects with atrophy of the hippocampus for FS and AA and their characteristics. FS, FreeSurfer; AA, ACM-Adaboost; HV, Hippocampal Volume; MMSE, Mini-Mental State Examination.*

We found a significant association of right and left hippocampus with age in FS (left: B −0.008, 95% Confidence Interval (CI) −0.010 to −0.007; right: B −0.009, 95% CI −0.012 to −0.008; *p* < 0.001) and AA (left: B −0.004, 95% CI −0.005 to −0.002; right: B −0.004, 95% CI −0.005 to −0.003; *p* < 0.001). Volume thresholds from 56 to 90 years are reported for FS and AA in Supplementary Tables 4, 5.

Table 3 shows that real hippocampal volumetry of the IBNA subjects were similar to those expected for subjects of the same age obtained from the ADNI population norms (*p* > 0.01).

**TABLE 3:** Comparison between real and computed volumes in IBNA subjects.

	n	V real	Percentile real	V computed	Percentile computed	Difference (95% CI)	*P*-value
**FS**							
FS lh	64	3638 ± 328	51st (20th–80th)	3,558 ± 192	42nd (24th–62nd)	80 (−10 to 170)	0.08
FS rh	64	3739 ± 340	48th (18th–78th)	3,688 ± 237	42nd (22nd–66th)	51 (−57 to 159)	0.35
**AA**							
AA lh	64	5314 ± 460	68th (26th–93rd)	5,294 ± 117	67th (56th–76th)	20 (−92 to 131)	0.73
AA rh	64	5376 ± 454	58th (18th–90th)	5,401 ± 151	60th (45th–74th)	−25 (−140 to 90)	0.67

*Volumes are reported in mm^3^ as means ± SD for both FS and AA. The “real” volumes are derived processing the IBNA MRIs with FS and AA tools. The “computed” volumes are derived from the GAMLSS models fitted from ADNI norms. In particular individual IBNA ages were entered in the model equations to compute the volumetric values. *P*-values were computed with *t*-test. n, sample size; FS, FreeSurfer; AA, ACM-Adaboost; V, Volume; CI, Confidence Interval.*

### Percentile Validation on ARWiBo

Table 4 presents the Italian ARWiBo cohort characteristics and results. ARWiBo was used as independent validation data set. For each diagnostic class (i.e., CN, MCI, AD) we considered 100 subjects. The AD subjects were older and less educated. AD subjects were more often ApoE ε4 carriers than MCI and CN. In the three diagnostic classes we observed a female gender preponderance. AD had higher CDR scores and lower MMSE compared to the other groups. As far as the neuropsychological tests are concerned, significant differences were found in all tests (*p* < 0.01). Comparability assessment of the subsamples used in each diagnostic class of Table 4 tested with multivariate statistics approach (*p* > 0.01) can be found as Supplementary Figure 6. Finally, we found significant differences in the hippocampal volumes computed by both pipelines (FS: *p* < 0.001; AA: *p* < 0.001). The *post-hoc* analyses revealed *p*-values less than 0.001 for each hippocampal volume comparison, as well.

**TABLE 4:** ARWiBo sociodemographic, clinical, neuropsychological, and morphological features.

	ARWiBo			*P*-value
	
	CN	MCI	AD	
Age	70 ± 8 (*n* = 100)	73 ± 6 (*n* = 100)	75 ± 7 (*n* = 100)	<0.001^€^
Education	10 ± 5 (*n* = 73)	8 ± 4 (*n* = 86)	7 ± 4 (*n* = 83)	<0.001^€^
ApoE4 carriers	4 (14%) (*n* = 29)	21 (32%) (*n* = 65)	21 (44%) (*n* = 48)	0.025^€^
Gender, females	51 (51%) (*n* = 100)	65 (65%) (*n* = 100)	73 (73%) (*n* = 100)	0.005^€^
Ethnicity	100% White (*n* = 100)	100% White (*n* = 100)	100% White (*n* = 100)	1
Hypertension	31 (44%) (*n* = 70)	34 (42%) (*n* = 80)	33 (45%) (*n* = 73)	0.943
Diabetes	10 (14%) (*n* = 70)	11 (14%) (*n* = 79)	7 (9%) (*n* = 74)	0.615
Heart disease	6 (9%) (*n* = 70)	20 (25%) (*n* = 80)	13 (18%) (*n* = 74)	0.030°
Severe obesity	0 (0%) (*n* = 63)	0 (0%) (*n* = 76)	0 (0%) (*n* = 46)	–
CDR	0 (92%) 0.5 (8%) 1 (0%) 2 (0%) 3 (0%) (*n* = 36)	0 (3%) 0.5 (90%) 1 (6%) 2 (0%) 3 (0%) (*n* = 63)	0 (0%) 0.5 (12%) 1 (76%) 2 (12%) 3 (0%) (*n* = 49)	<0.001^€^ < 0.001^§^ < 0.001^€§^ – –
Depression*	0 [−0.26; +0.26] (*n* = 59)	0.36 [0.00; +0.73] (*n* = 77)	0.08 [−0.22; +0.40] (*n* = 63)	0.075
MMSE	0 [−0.29; +0.29] (*n* = 45)	−0.97 [−1.22; −0.72] (*n* = 92)	−3.73 [−4.25; −3.21] (*n* = 87)	<0.001°^€§^
TMT-A	0 [−2.06; +2.06] (*n* = 25)	9.24 [+4.93; +13.55] (*n* = 38)	20.53 [+14.66; +26.41] (*n* = 30)	<0.001°^€§^
TMT-B	0 [−2.08; +2.08] (*n* = 22)	11.91 [+7.14; +16.67] (*n* = 36)	11.43 [+5.94; +16.92] (*n* = 16)	<0.001°^€^
TMT B-A	0 [−2.08; +2.08] (*n* = 22)	9.76 [+5.38; +14.14] (*n* = 36)	8.12 [+3.17; +13.07] (*n* = 16)	0.002°^€^
Verbal fluency (phonemic)	0 [−0.34; +0.34] (*n* = 33)	−0.71 [−1.02; −0.40] (*n* = 38)	−1.48 [−1.73; −1.23] (*n* = 41)	<0.001°^€§^
Verbal fluency (semantic)	0 [−0.34; +0.34] (*n* = 33)	−1.27 [−1.58; −0.98] (*n* = 38)	−2.33 [−2.50; −2.17] (*n* = 42)	<0.001°^€§^
Clock drawing test	0 [−2.37; +2.37] (*n* = 8)	6.29 [+4.42; +8.16] (*n* = 71)	14.62 [+12.69; +16.55] (*n* = 58)	<0.001°^€§^
Left FS HV	3,530 ± 374 (*n* = 100)	3,181 ± 429 (*n* = 100)	2,872 ± 371 (*n* = 100)	<0.001°^€§^
Right FS HV	3,635 ± 421 (*n* = 100)	3,270 ± 461 (*n* = 100)	2,963 ± 409 (*n* = 100)	<0.001°^€§^
Left AA HV	5,027 ± 597 (*n* = 100)	4,647 ± 599 (*n* = 100)	4,114 ± 535 (*n* = 100)	<0.001°^€§^
Right AA HV	5,162 ± 553 (*n* = 100)	4,723 ± 583 (*n* = 100)	4,298 ± 569 (*n* = 100)	<0.001°^€§^

*Features of the Italian ARWiBo data set selected. Values denote means ± standard deviation or number (%). The 95% Confidence Intervals (CI) of z-scores or t-scores (when at least one group size was *n* < 30) are reported in square brackets. *P*-values are obtained with Chi-square test for dichotomous and Kruskal-Wallis test for continuous variables.*

**Post-hoc* analysis: °significant difference between CN and MCI.*

*^€^Significant difference between CN and AD.*

*^§^Significant difference between MCI and AD.*

ARWiBo, Alzheimer’s disease Repository Without Borders; n, sample size; CN, Cognitively intact healthy control; MCI, Mild Cognitive Impairment; AD, Alzheimer’s Dementia; CDR, Clinical Dementia Rating scale; MMSE, Mini-Mental State Examination; TMT-A, Trail Making Test (part A); TMT-B, Trail Making Test (part B); TMT B-A, Trail Making Test (part B minus part A); HV, Hippocampal Volume.

*Brief Symptom Inventory (BSI) scale.

For sake of completeness, the comparison among ARWiBo and both IBNA and ADNI hippocampal volumes were investigated and *p*-values reported in Supplementary Table 6. Furthermore, the inter-subject variability was not significant among the three matched data sets of controls (see Supplementary Figure 7).

The influence of the years of education, gender, and ApoE4 status (considering only subjects without missing values) were reported in Supplementary Table 7. GLM results concerning the effect of field strength and vendors are reported in Supplementary Table 8.

**FIGURE 2:**
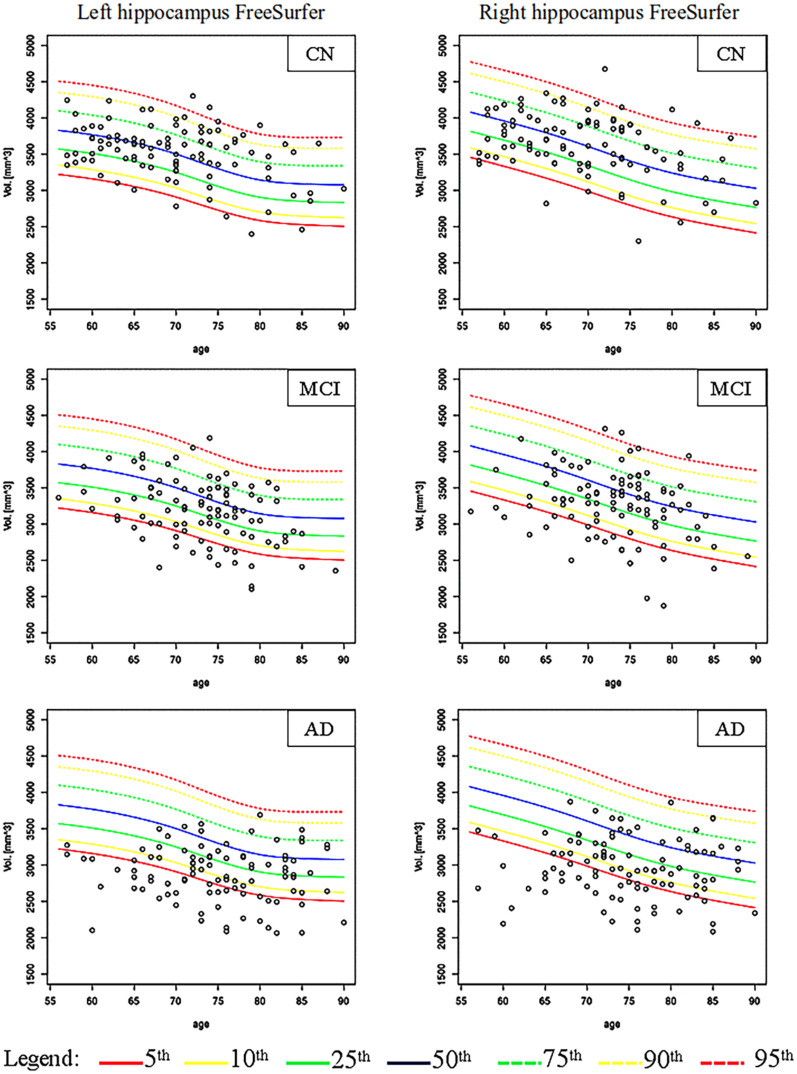
Age-specific percentile distribution of ARWiBo hippocampal volumes processed with FS. Left and right scatter plots of ARWiBo hippocampal volumes (mm^3^) (100 CN, 100 MCI, 100 AD) from 56 to 90 years on the ADNI percentiles chart. CN, cognitively intact healthy Control; MCI, Mild Cognitive Impairment; AD, Alzheimer’s Dementia.

**FIGURE 3:**
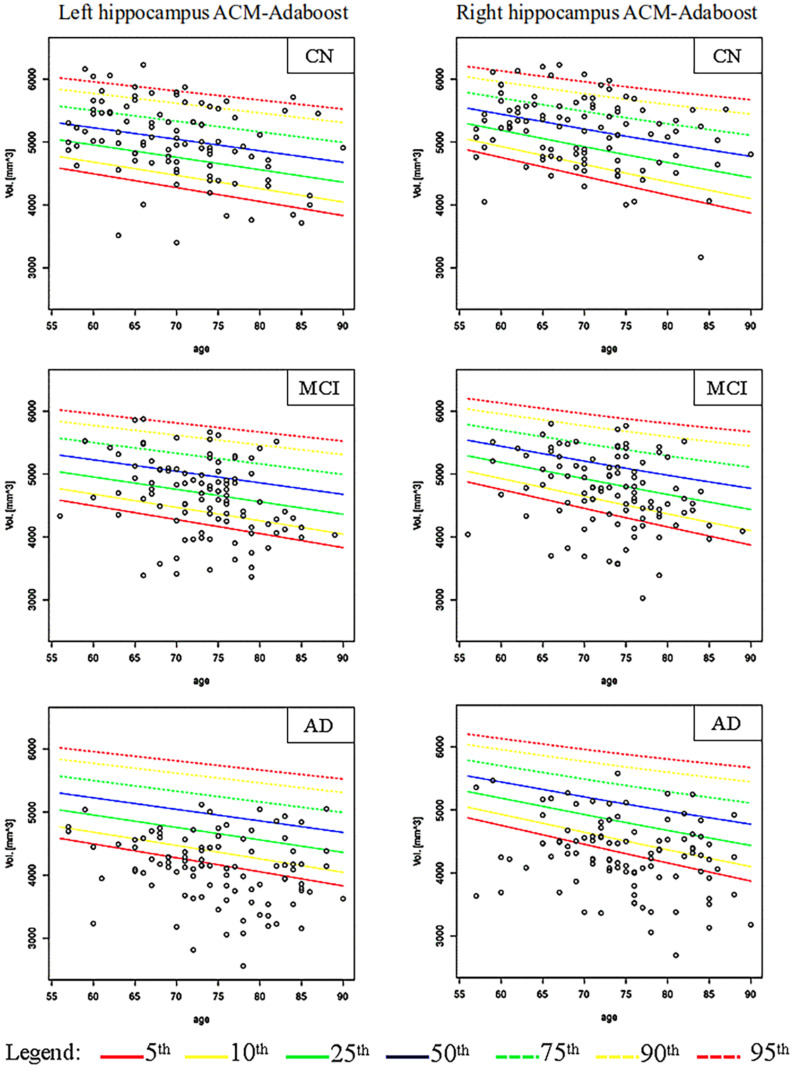
Age-specific percentile distribution of ARWiBo hippocampal volumes processed with AA. Left and right scatter plots of ARWiBo hippocampal volumes (mm^3^) (100 CN, 100 MCI, 100 AD) from 56 to 90 years on the ADNI percentiles chart. CN, cognitively intact healthy Control; MCI, Mild Cognitive Impairment; AD, Alzheimer’s Dementia.

Figures 2, 3 show the scatter plots of ARWiBo subjects processed with FS and AA, respectively. CN covered all the percentile curves with a mean hippocampal volume close to the 50th; the MCI subgroup were close to the 25th percentile; while AD subgroup volumes fell around the 10th percentile for FS and the 5th for AA (Table 5). Cohen’s κ correlation coefficient of the same ARWiBo subjects below the 5th percentile analyzed with both FS and AA was equal to 0.51 for left and 0.49 for right hippocampus.

**TABLE 5:** Mean hippocampal volume percentiles of the ARWIBO cohort.

	CN		MCI		AD	
			
	FS	AA	FS	AA	FS	AA
Left HV	52th	48th	30th	22th	11th	4th
Right HV	52th	45th	29th	17th	12th	5th

*Values denote the percentile curve where the mean hippocampal volume of each diagnostic class and pipeline is. FS, FreeSurfer; AA, ACM-Adaboost; HV, Hippocampal Volume; CN, Cognitively intact healthy control; MCI, Mild Cognitive Impairment; AD, Alzheimer’s Dementia.*

Finally, Table 6 shows the Sensitivity (Se), Specificity (Sp), Positive Predictive Value (PPV), Negative Predictive Value (NPV) metrics of diagnostic accuracy and the Chi-squared test used to evaluate the discrepancy between the percentage of subjects under the 5th percentile and over the 95th percentile compared to the expected values. We considered the 100 CN individuals of the ARWiBo data set.

**TABLE 6:** Accuracy metrics of FS and AA at identifying ARWiBo abnormal subjects.

	FS				AA			
		
	LH (*n* = 100)		RH (*n* = 100)		LH (*n* = 100)		RH (*n* = 100)	
				
	<5th percentile	>95th percentile	<5th percentile	>95th percentile	<5th percentile	>95th percentile	<5th percentile	>95th percentile
Se	1	0.60	1	0.80	1	1	1	1
Sp	1	1	0.99	1	0.98	0.99	0.97	0.99
PPV	1	1	0.83	1	0.71	0.83	0.63	0.83
NPV	1	0.98	1	0.99	1	1	1	1
Percentage (*P*-values)	5% (1)	3% (0.359)	6% (0.646)	4% (0.646)	7% (0.359)	6% (0.646)	8% (0.169)	6% (0.646)

*Accuracy metrics used to determine the fitness of FS and AA at identifying ARWiBo abnormal subjects and comparison of cases under the 5th and above the 95th percentiles for the 100 CN from the independent ARWiBo data set. Percentages were computed counting those subjects whose hippocampal volumes were below the 5th and above the 95th ADNI percentiles. *P*-values were computed as Chi-squared test. FS, FreeSurfer; AA, ACM-Adaboost; n, sample size; LH, Left hippocampus; RH, Right hippocampus; Se, sensitivity; Sp, specificity; PPV, positive predictive value; NPV, negative predictive value.*

## Discussion

This study shows that it is possible to define the norms originated from a large number of ADNI high-resolution brain T13D MPRAGE images and to apply them into a clinical routine application (i.e., as a supportive biomarker for AD diagnosis) on the Italian population. These percentiles can reasonably be used as a reference for judgment of structural normality in patients with cognitive impairment of suspected AD through a single-case medial temporal atrophy (MTA) analysis. Several findings of the present study deserve specific comment.

### Segmentation Algorithms

The hippocampal volume measured with FS is systematically lower by one third if compared to AA’s. Explanations for this evidence are related to the different mathematical procedures used by the two tools when segmenting. FS classifies the MRI voxels using a probabilistic atlas, AA learns classification rules from hippocampal region based on intensity, positional and morphological features. An important role is also played by the different segmentation protocols adopted by the two algorithms. FS pries on a manual segmentation protocol developed by experts at MGH. In contrast, AA is based on EADC-ADNI HarP protocol. These differences contribute to explain the adoption of two slightly different monotonic descendant functions, such as Gamma for FS and Logistic for AA, in the percentiles fitting. The volumes of the hippocampi segmented with the MGH and HarP were found to be highly correlated with Tau, Amyloid-β burden, and the Braak staging in AD. This demonstrates that both protocols can capture AD-related pathologies with good evidence of validity (Stricker et al., 2012; Frisoni et al., 2015).

In both pipelines, right hippocampal volumes were higher than left ones in agreement with the literature data. These evidences can additionally be taken as indirect proof of the accuracy of our volumetric measurements. Our results showed that the hippocampal volumes decline progressively after 56 years. Walhovd et al. (2011) and Fjell et al. (2013) reported comparable results. We found also a significant association between HV and age as reported in many studies (Good et al., 2001; Grieve et al., 2005; Knopman et al., 2016).

Nevertheless both FS’s and AA’s performances varied, pointing out that neither algorithm can be considered as more effective. AA reduces the computational cost of 10 h on average compared to FS (i.e., ≅11.5 h per single subject in FS; ≅1.0 h per single subject in AA) which, however, is less error-prone. This is due to the fact that FS performs the so-called “estimated” computation of the TIV by exploiting its correlation with the determinant of the transform matrix obtained from the Talairach registration (Buckner et al., 2004), while AA uses SPM routines (Malone et al., 2015) where the TIV is computed adding the volumes of CSF, gray matter and white matter obtained from the brain tissue segmentation. Therefore, it is clear that AA must be accurate in two complex routines (i.e., hippocampal and TIV segmentations) that unavoidably affect its final success rate resulting most likely in higher Type I error or False Positive rate than FS. In light of this, it may be appropriate to consider concomitantly the results obtained from the two pipelines and, eventually, choose one tool or the other according to the specific end-user’s needs (e.g., time urgency, hippocampal segmentation protocol preferences, specificity thresholds).

### American and Italian Data Sets Comparison

An important requirement in defining a normative population is the absence of selection bias. Indeed, in our study there were few issues in the selection of the normative population. One was that ADNI normative subjects used to derive the percentiles were not randomly drawn from the general population. Moreover, ADNI is a US observational study with specific selection criteria. For those reasons we compared a well-characterized subgroup of ADNI subjects and features with a data set representative of the Italian general population (IBNA) and we further evaluated the norms with an external validation data set, i.e., the ARWiBo cohort.

The lack of significant difference in clinical, neuropsychological and morphometric features among IBNA and ADNI suggested the feasibility of this comparison. Given the strong effect of physical health on cognitive function in older persons, it was necessary to check that related features (e.g., hypertension, diabetes, heart disease, obesity) in ADNI groups were overall similar to those of the Italian general population.

Among the neuropsychological tests assessed, no differences in performance were observed. In particular, lack of MMSE differences were indicative of normal and comparable global cognition in both IBNA and ADNI. Furthermore, the performances on the attention and mental speed (TMT-A and B), executive control abilities (TMT B-A, clock drawing), memory (logical memory, digit span, Rey auditory verbal learning), and language (verbal fluency) of the US population compared to the Italian one were analogous.

The morphometric data of IBNA were also similar to the ones we expected from the age-based model built on the norms created from ADNI. This additional evidence indicates that the characteristics of the ADNI study fitted well the Italian general population.

### Percentile Validation

The Chi-squared test showed the conformity of the AA and FS data volume distributions to the expected ones. We performed discrepancy tests with good results. Both algorithms had high *p*-values confirming the null hypothesis and indicating that the percentiles fitted the data well. For the right hippocampus, AA exhibited a higher percentage of CN below the 5th percentile: respectively 8 vs. 6% of FS. While for the left hippocampus the percentages were 7% for AA vs. 5% of FS. To validate the percentile reference charts, the large independent data set of ARWiBo Italian subjects were plotted against the ADNI norms too. The average volume of each diagnostic class resulted consistent with the predicted progression of hippocampal atrophy in the AD. The two algorithms showed a moderate agreement in the classification of the same ARWiBo subjects under the 5th percentile.

### Hippocampal Atrophy Norms for Italian General Population

The definition of norms is strategic in the context of clinical setting, especially at this point in time in which consolidated brain acquisition standards and harmonized data sets, with thousands of T13D brain images publicly collected in e-infrastructures, such as LONI (Crawford et al., 2016) and neuGRID (Frisoni et al., 2011; Redolfi et al., 2013, 2015), are available. Second, these algorithms are capable to provide reliable measurements (Boccardi et al., 2011; Khlif et al., 2019) without the requirement of expert tracers. The possibility to use automatic and accurate segmentation tools represent a giant step forward reducing the operator inter/intra-subjective errors during the manual tracing and improving the replicability of the final results. Moreover the correlation of the manual segmentation, considered the gold standard, with the automatic segmentation pipelines has been demonstrated revealing good similarities, despite AdaBoost method generally correlated higher than FreeSurfer (Schmidt et al., 2018; Khlif et al., 2019).

The morphometric data reported in this study (Supplementary Tables 4, 5) may serve as norms for comparison with morphological brain changes associated with AD. In particular, reduced hippocampal volume is a sensitive marker of AD progression and it is included in the NIA and Internal Working Group (IWG-2) diagnostic criteria (Dubois et al., 2014).

Recently, many Italian initiatives arose with a special focus on the diagnosis of the preclinical or “prodromal” stage of AD, when symptoms are still absent or very mild, in order to start a pharmacological intervention capable of slowing down the disease progression. At the time of writing, through an exploration of the “Clicaltrial.gov” database^6^, we found nine on-going observational and clinical studies in Italy. Among these, the INTERCEPTOR study (Rossini et al., 2019)^7^ aims at identifying those biomarkers that allow the best prediction of conversion of individuals at risk of developing AD. A conspicuous number of volumetric T13D MRI scans has been collected for the assessment of the hippocampal atrophy via the MTA single-case analysis. In such scenarios is essential to have in place precise hippocampal volumetric measurements to support diagnosis with the objective to assess the efficacy of candidate disease-modifying treatments or interventions on modifiable life-style risk factors. The norms generated in the present study might be used as cut-off to define the progression of the disease and may be included in national standard operative procedure to monitor the departure from normal cognitive aging.

### Limitations

Some methodological limitations of the present study should be acknowledged.

ARWiBo cohort presented diagnostic classes with some group heterogeneity. In detail, MCI were amnestic or non-amnestic with single or multi domain. In AD there were probable, possible, and mixed clinical variants.

Our GAMLSS models did not take into consideration either sex or magnetic field strength or scanner manufacturer predictors that marginally influence the estimations of the final norms. Sex influence had discrepancies between brain regions and diminishes with age. Recent findings suggest there is no substantial difference between men and women after correcting for TIV (Potvin et al., 2016). As far as field strength and MRI vendors are concerned, there are also evidences that the influence of these characteristics on the hippocampal volumes remain very modest (Potvin et al., 2016; Whelan et al., 2016; Quattrini et al., 2020). Potvin et al. (2016) revealed that the influence of magnetic field strength and manufacturer is very small on the whole hippocampus respect to other variables. Whelan et al., 2016 disclose that FS version 6.0 produced consistent estimates of the hippocampal volume across lower (1.5 T) and higher (3 T) MRI scanner field strengths finding an intraclass correlation coefficient of 0.94. Quattrini et al., 2020 also assessed the reliability of the automated segmentation of the hippocampus in 13 sites and 3 different scanner manufacturers, revealing for the whole hippocampus a reproducibility error less than 5%. All the GLM analyses we conducted were in line with these results where the aforementioned factors influenced only weakly the hippocampal volume of our cohorts.

The prevalence of the ApoE4 allele in ADNI subjects was higher than that reported in the Italian community-based populations of IBNA. This mismatch was expected because the ApoE4 allele frequency is normally influenced by several well-known factors (Kern et al., 2015), such as region of origin, ethnicity, and sex. Therefore, although it represents a greater risk factor for the ADNI subjects, potentially undermining their future cognitive reserve, however, at time of MRI acquisition they were clinically labeled as CN without MTA.

One should also note that the normative sample used was just cross-sectional without spanning the longitudinal information of each individual. *Ex post* evidences revealed that 22% of ADNI subjects used had not follow up information; while 69% remained stable and healthy over the next 48 months after the initial assessment. Although the great majority remained stable, 9% of ADNI subjects converted with a different pace to AD (the conversion time in average was 65 months). In future studies we should better refine the normative group using with attention the follow-up information as well.

### Future Developments

We are confident this study will represent a step forward for the adoptability of a common Italian normative reference against which to compare new individuals from clinical populations. However, in addition to these promising results, future efforts should clarify the ability of FS and AA norms to: (i) track consistently the individual hippocampal decline along consecutive follow ups, (ii) identify much earlier the subjects at higher risk of progression, (iii) help monitoring the efficacy of future disease modifying drugs.

## Conclusion

The present study is the first attempt to generate accessible and fully automatic brain hippocampal norms in Italian adults. The subjects selected from ADNI study had neuropsychological, morphometric and clinical features consistent with those of the Italian general population. These percentiles can be used as a reliable reference for Italian subjects with suspected AD, thus allowing single-case analysis. FS and AA reports generation is publicly available via neuGRID platform. The generated results are meant to be reused by other upcoming national neuroimaging research groups.

## Data Availability Statement

The datasets presented in this study can be found in online repositories. The names of the repository/repositories and accession number(s) can be found below: data used in preparation of this article were obtained from the Alzheimer’s Disease Repository Without Borders (ARWiBo—www.arwibo.it). ARWiBo is publicly accessible via neuGRID platform (https://www.neugrid2.eu). ADNI is publicly accessible via the web-portal of the Laboratory of NeuroImaging (LONI) (http://adni.loni.usc.edu). The pipelines (FS v.6.0 and AA) used in the study are publicly accessible via neuGRID platform (https://www.neugrid2.eu).

## Ethics Statement

Ethical review and approval was not required for the study on human participants in accordance with the local legislation and institutional requirements. The patients/participants provided their written informed consent to participate in this study.

## Author Contributions

SD: formal analysis, investigation, software, data curation, visualization, validation, and writing. SG: methodology and review—editing. NV: methodology, investigation, project administration, and review—editing. CF: formal analysis and review—editing. PMR and SFC: resources, project administration, and review—editing. GBF: conceptualization, methodology, resources, and supervision. AR: conceptualization, methodology, resources, formal analysis, data curation, writing, and supervision. All authors contributed to the article and approved the submitted version.

## Conflict of Interest

The authors declare that the research was conducted in the absence of any commercial or financial relationships that could be construed as a potential conflict of interest.

## Footnotes

1 www.neugrid2.eu

2 http://adni.loni.usc.edu

3 http://adni.loni.usc.edu/methods/documents/mri-protocols/

4 www.hippocampal-protocol.net

5 https://www.fil.ion.ucl.ac.uk/spm/

6 https://clinicaltrials.gov/

7 www.interceptorproject.com/en/

## Abbreviations

AA: ACM-Adaboost
AD: Alzheimer’s Dementia
ADAS-Cog: Alzheimer’s Disease Assessment Scale—Cognitive Subscale
ADNI: Alzheimer’s Disease Neuroimaging Initiative
ANOVA: analysis of variance
ApoE4: Apolipoprotein E ε 4
ARWiBo: Alzheimer’s disease Repository Without Borders
BMI: Body Mass Index
BSI: Brief Symptom Inventory
CDR: Clinical Dementia Rating
CI: Confidence Interval
CN: cognitively intact healthy controls
eTIV: estimated TIV
FS: FreeSurfer
HV: hippocampal volume
GAMLSS: Generalized Additive Models for Location, Scale and Shape
GDS: Geriatric Depression Scale
GLM: Generalized Linear Model
IBNA: Italian Brain Normative Archive
IDA: Imaging Data Archive
ICBM: International Consortium for Brain Mapping
ILSA: Italian Longitudinal Study on Aging
IWG: Internal Working Group
MCI: mild cognitive impairments
MGH: Massachusetts General Hospital
ML: machine learning
MMSE: Mini Mental State Examination
MoCA: Montreal Cognitive Assessment
MPRAGE: magnetization-prepared rapid acquisition with gradient echo
MRF: Markov Random Field
MRI: Magnetic Resonance imaging
MTA: medial temporal atrophy
NPV: Negative Predictive Value
LONI: Laboratory of NeuroImaging
NIA-AA: National Institute on Aging and Alzheimer’s Association
PACS: Picture Archiving and Communication Systems
PPV: Positive Predictive Value
SD: Standard Deviation
Se: Sensitivity
Sp: Specificity
SPM: Statistical Parametric Mapping
T13D: T1-weighted 3D
TIV: intracranial volume
TMT: Trail Making Test
UCLA: University of California Los Angeles.
