# Supplementary figures and images for: Norms for Automatic Estimation of Hippocampal Atrophy and a Step Forward for Applicability to the Italian Population
Source: Front Neurosci. 2021 Jun 28;15:656808. doi: 10.3389/fnins.2021.656808 (PMC8273578; doi:10.3389/fnins.2021.656808)

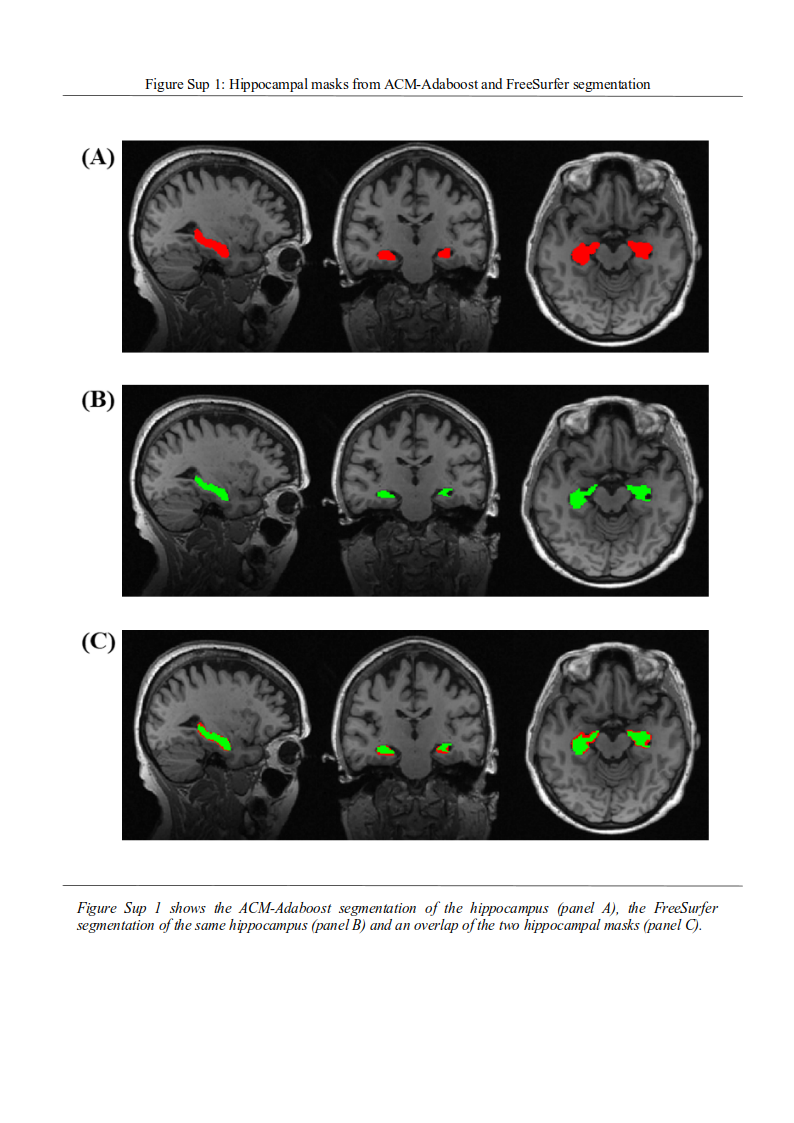

Supplement: Supplementary file 1 [file Image_1.PNG]

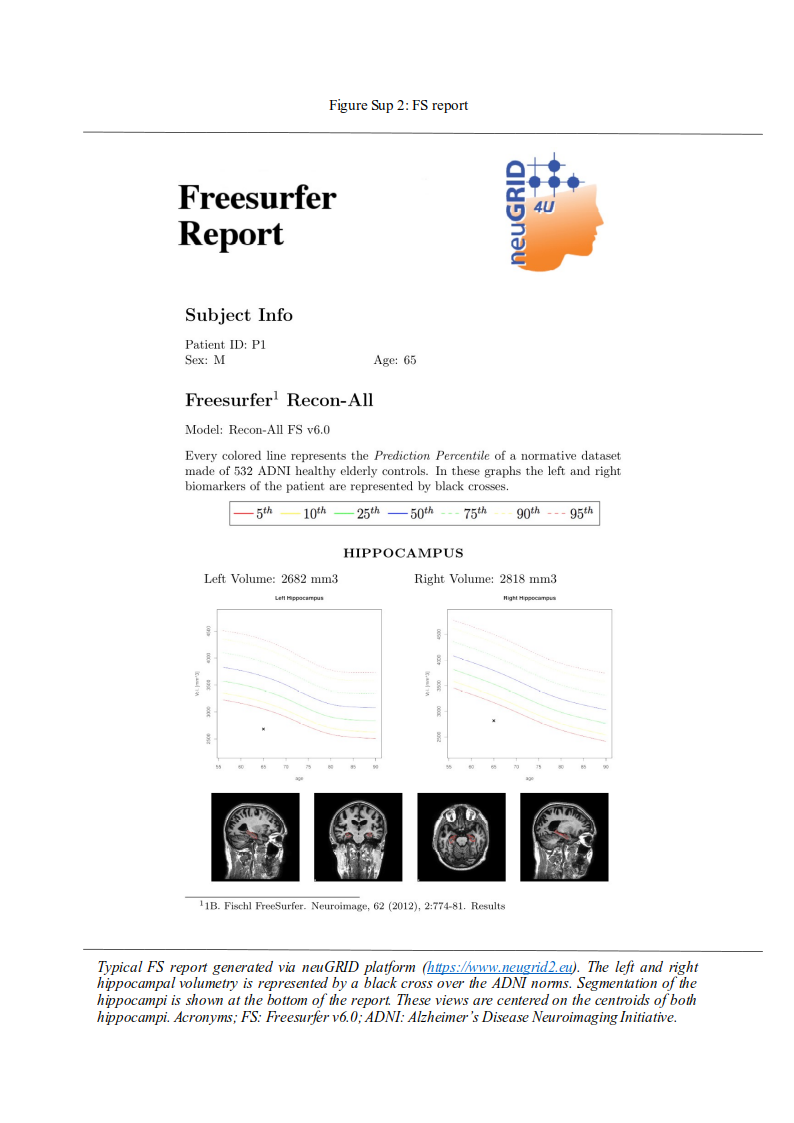

Supplement: Supplementary file 2 [file Image_2.PNG]

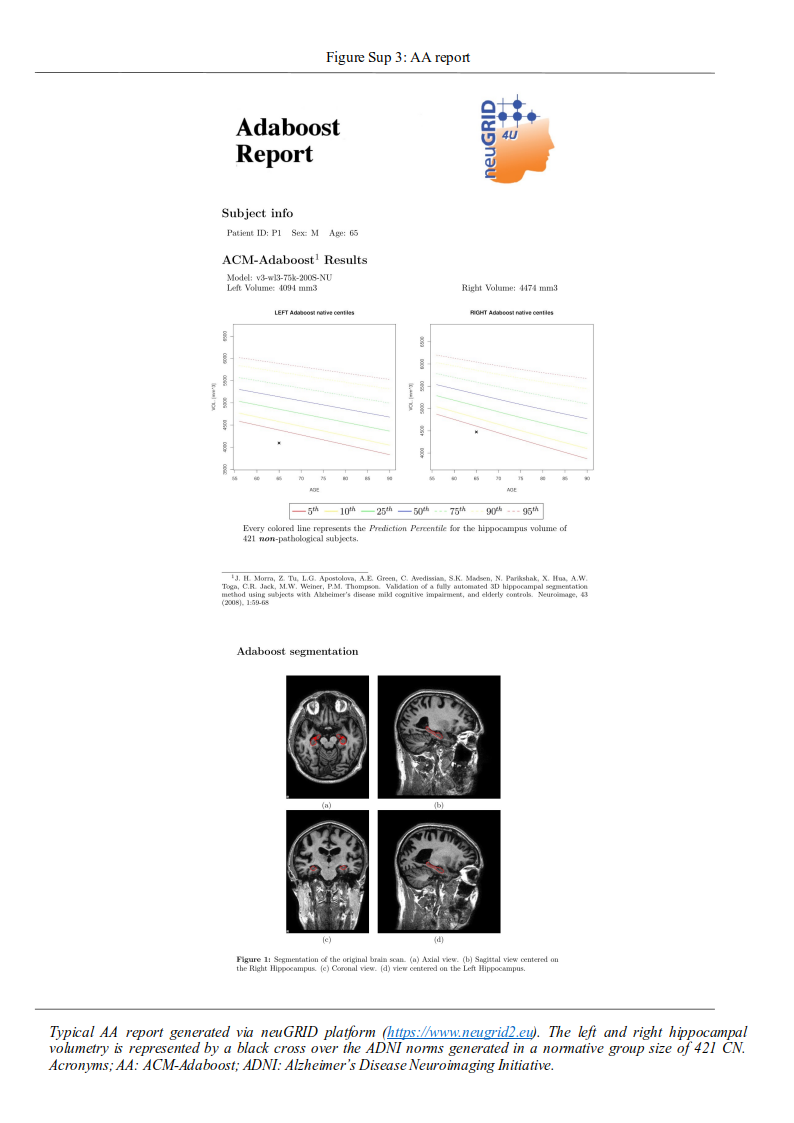

Supplement: Supplementary file 3 [file Image_3.PNG]

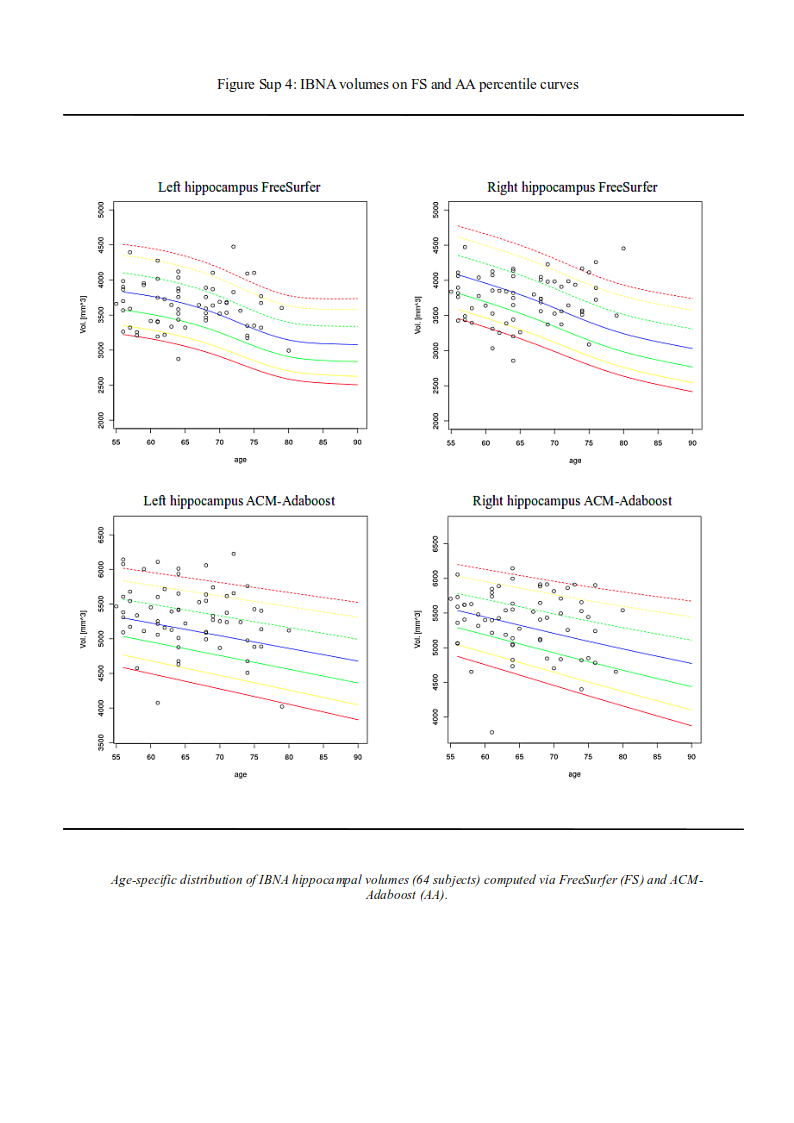

Supplement: Supplementary file 4 [file Image_4.PNG]

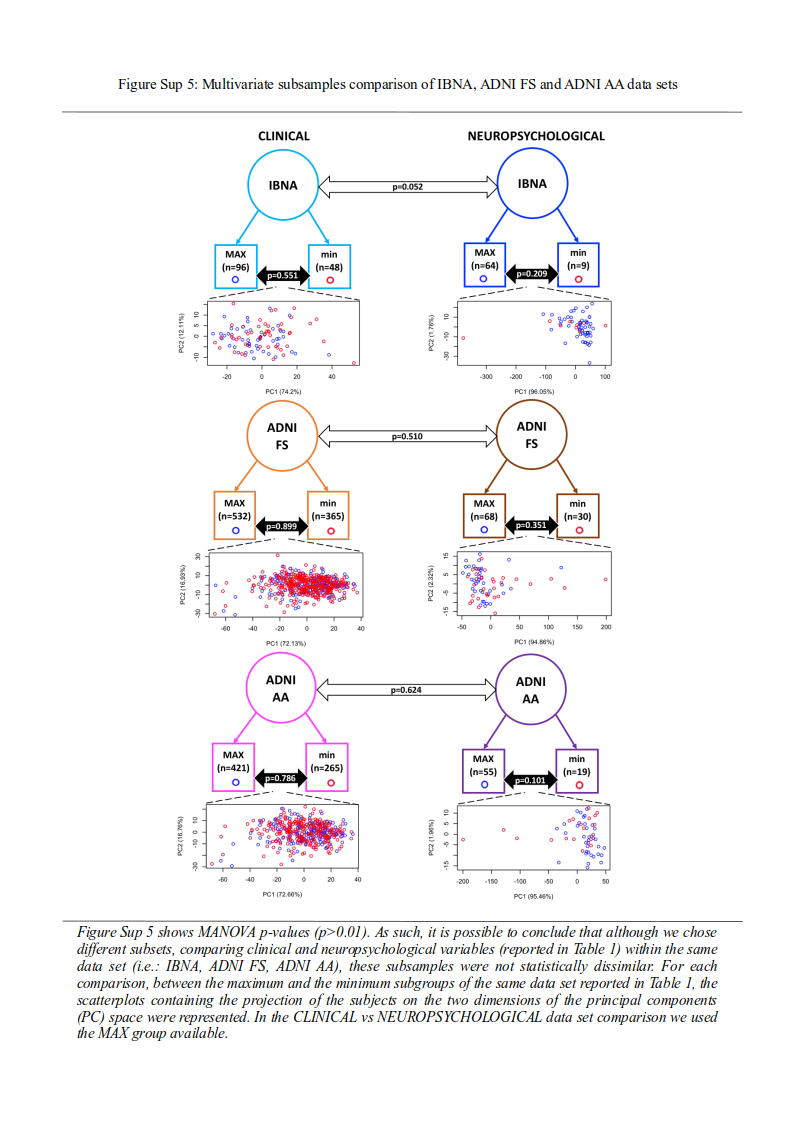

Supplement: Supplementary file 5 [file Image_5.PNG]

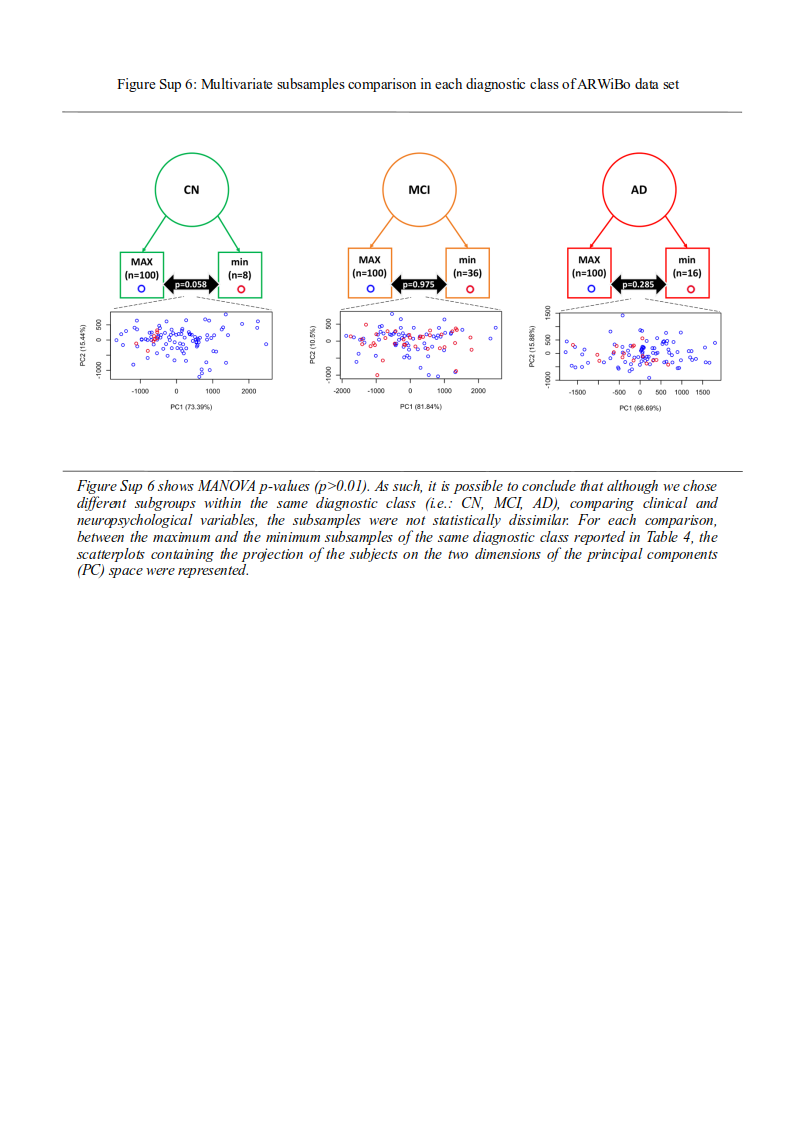

Supplement: Supplementary file 6 [file Image_6.PNG]

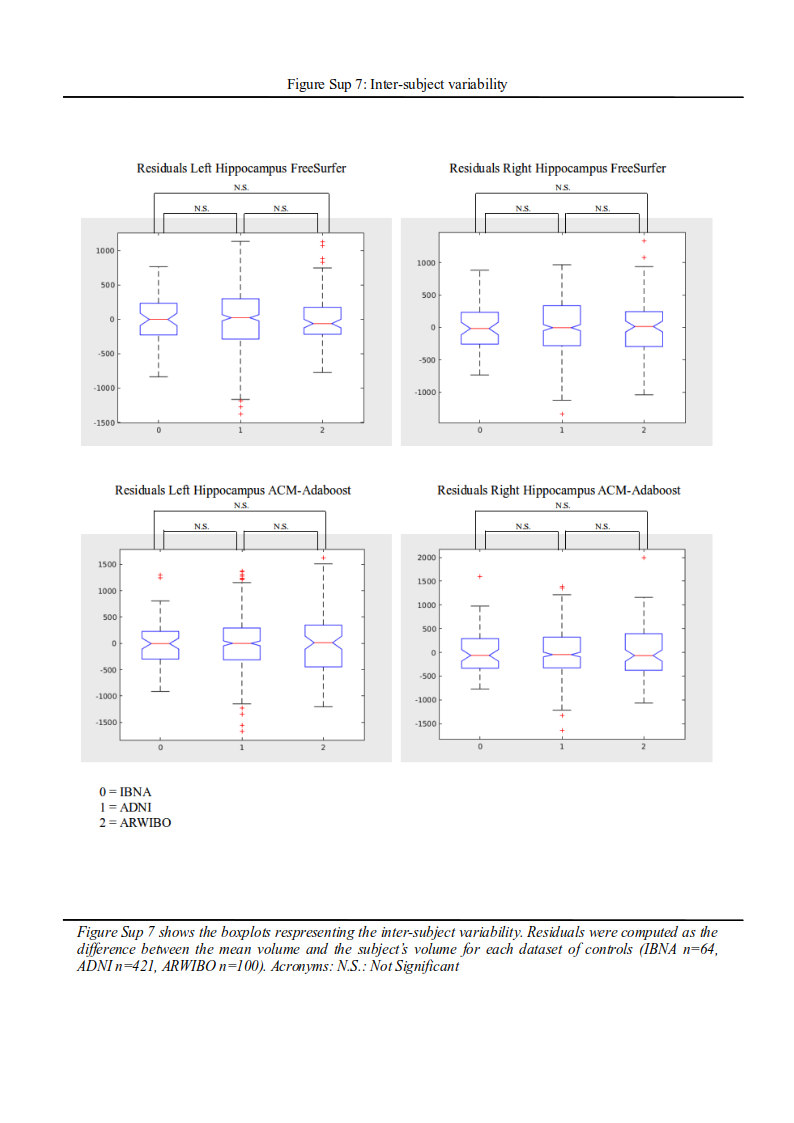

Supplement: Supplementary file 7 [file Image_7.PNG]
